# Valuing Chinese medicine quality of life-11 dimensions (CQ-11D) health states using a discrete choice experiment with survival duration (DCE_TTO_)

**DOI:** 10.1186/s12955-023-02180-4

**Published:** 2023-08-23

**Authors:** Wentao Zhu, Mengpei Zhang, Jie Pan, Lizheng Shi, Hailiang Gao, Shitong Xie

**Affiliations:** 1 Beijing University of Chinese Medicine, Higher Education Zone in LiangXiang Town, FangShan District, Beijing, 102488 China; 2 Tulane University, 1440 Canal Street Suite 1900, New Orleans, LA 70112 USA; 3 School of Pharmaceutical Science and Technology, Tianjin University, Tianjin, 300072 China

**Keywords:** CQ-11D, DCE_TTO_, Health utility, Value set, Chinese population

## Abstract

**Objective:**

To explore generating a health utility value set for the Chinese medicine Quality of life-11 Dimensions (CQ-11D), a utility instrument designed to assess patients’ health status while receiving TCM treatment, among the Chinese population.

**Methods:**

The study was designed to recruit at least 2400 respondents across mainland China to complete one-to-one, face-to-face interviews. Respondents completed ten discrete choice experiment with survival duration (DCE_TTO_) tasks during interviews. The conditional logit models were used to generate the health utility value set for the CQ-11D using the DCE_TTO_ data.

**Results:**

A total of 2,586 respondents were invited to participate in the survey and 2498 valid interviews were completed (a completion rate of 96.60%). The modified conditional logit model with combing logically inconsistent levels was ultimately selected to construct the health utility value set for the CQ-11D instrument. The range of the measurable health utility value was -0.868 ~ 1.

**Conclusion:**

The study provides the first utility value set for the CQ-11D among the Chinese population. The CQ-11D and corresponding utility value set can be used to measure the health utility values of patients undergoing traditional Chinese medicine interventions, and further facilitate relevant cost-utility analyses. The application of the CQ-11D can support TCM resource allocation in China.

**Supplementary Information:**

The online version contains supplementary material available at 10.1186/s12955-023-02180-4.

## Introduction

Economic evaluations of health care interventions often involve incremental cost-effectiveness ratios, where the quality-adjusted life-year (QALY) is used to capture the health outcome of different interventions. Generic preference-based measures (GPBMs) are commonly used to calculate the QALY. Most of the GPBMs, such as the EQ-5D and SF-6D, were developed in Europe and North America, and are often translated into other languages to use in many non-English speaking countries [1]. One of the advantages of using these international GPBMs is that researchers can use the same instrument to measure the health-related quality of life (HRQoL) of populations in different counties or regions, allowing for cross-country/cross-cultural comparisons [2]. Similarly, when these GPBMs are applied in China for assessing health outcomes associated with Traditional Chinese Medicine (TCM), researchers can apply the adapted versions with their corresponding health utility value sets generated based on the Chinese population.

However, health is a culturally related concept, and health evaluation indicators formulated in the Western cultural environment may not include Chinese cultural views on health. A study evaluating the similarities and differences of health-related quality of life concepts between the East and the West compared 8 HRQoL instruments developed in the Chinese cultural context with 3 HRQoL instruments developed outside China. This study found that, although there is a consensus between the East and West on some of the HRQoL domains, domains such as emotional control, weather adaptation, social adaptation, spirituality, and skin color are unique to the Chinese cultural background [3]. Mao Z et al. [4] conducted a Q-methodological investigation study, and the results showed that several HRQoL domains were rated highly as most important by a diverse range of Chinese respondents but were not covered in the commonly used Western HRQoL instrument, such as the EQ-5D.

**Fig. 1:**
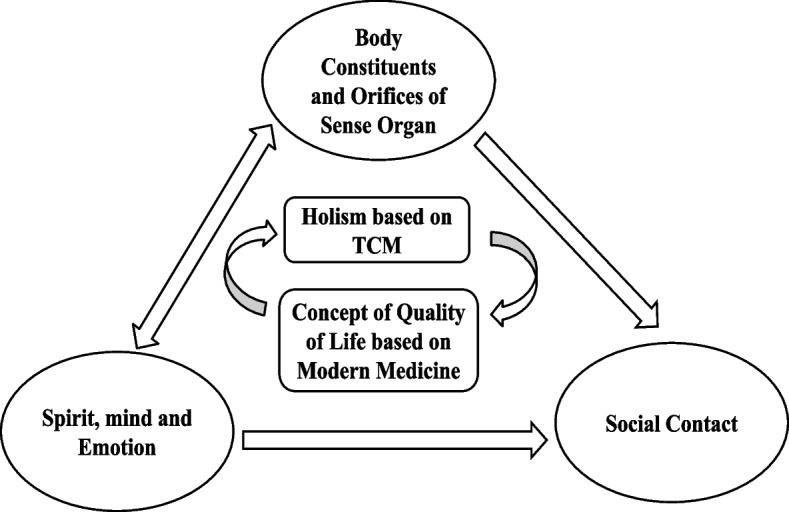
Theoretical framework of CQ-11D

Traditional Chinese medicine is a model related to the concept of health in Chinese traditional culture, which better reflects the understanding of Chinese culture on health. Some HRQoL instruments developed in China, such as the Chinese quality of life scale [5, 6], the Chinese PRO scale [7], and the sub-health assessment scale [8], have designed health indicators including spirit, appetite, sleep, and other concepts, and have been widely used among the Chinese population. These instruments all reflect the relevant domains of TCM health concept such as "unity of body and spirit, unity of man and nature, unity of man and society," "seven emotions", and "shape, spirit, and emotion." In brief, well-rounded health is the unity of inseparability of the body (including orifices of sense organs) and spirit (including emotion and mind), adaptation to the natural environment and society as well as the harmony of social contact. In TCM terms, the body is an outward manifestation of the spirit, and the spirit is the master of the body. Therefore, the coordination between body and spirit [9] and correspondence between the natural environment and the human body constitute the TCM holism that maintains the consistency of the bio-psycho-social medical model (Fig. 1). However, there are no items with similar meanings as these concepts in the international GPBMs such as the EQ-5D. Therefore, the health states described by these international instruments may not be consistent with TCM theories [10–13], and therefore, might not be comprehensive for evaluating TCM treatments. Besides, it is commonly recognized that the EQ-5D is not sensitive enough for assessing sub-health conditions due to its ceiling effects, whereas the SF-6D is not adequate for discriminating mild diseases [14, 15].

The CQ-11D (Chinese Medicine Quality of life-11 Dimensions) was therefore developed by the Zhu WT et al. at the Institute of Pharmacoeconomic Evaluation of Chinese Medicine from Beijing University of Chinese Medicine in 2021. The CQ-11D was developed based on the optimization of the first version of the Chinese Medicine QOL assessment scale (CM-QOL), with the overall view of traditional Chinese medicine and the health concept as the guiding ideology, using literature research, patient interviews, expert consultation, questionnaire surveys and constructed through standard processes. CM-QOL includes 19 items, such as complexion, appetite, sleep quality, stool, and attention. To make the original scale more suitable for compiling discrete choice experiment (DCE) tasks to develop a health utility value set, the CQ-11D was developed by modifying the items included in the CM-QOL. This proves was referred to a previous study modifying the SF-36 to the SF-6D conducted by Brazier et al. [16]. The basic principles of the modification are the followings: ^①^To avoid the redundancy of entries, if there are two or more items that basically describe the same aspect of health and are closely related, then only one entry is kept; ^②^Items with negative descriptions are preferentially reserved because these items are considered more relevant to health assessments and services. After modification, a TCM HRQoL instrument, the CQ-11D, with 11 items and four levels for each item was finally developed. After evaluations of the measurement properties, it was demonstrated that the CQ-11D has good reliability, construct validity, and standard correlation validity [17]. The CQ-11D has been issued by the China Association of Chinese Medicine under the standard number T/CACM1372-2021 with a release date of August 18, 2021, and an implementation date of August 18, 2021 [18].

DCE with survival duration (DCE_TTO_) is a relatively new preference elicitation technique that is successfully used to generate health utility value sets for GPBMs in many different countries [19–25]. This technique has not been used previously to value a TCM HRQoL instrument. Respondents complete a series of choice sets, including health state descriptions with an corresponding survival duration. Responses are modeled to generate a set of coefficients that lying on the 1–0 full health–dead QALY scale to calculate the utility values of all health states described by the classification system [26].

Since TCM plays an important role as a kind of Complementary and Alternative Medicine (CAM) for healthcare systems worldwide, a validated instrument for assessing disease impacts and health outcomes is needed for TCM interventions. We aimed to develop the health utility value set for the CQ-11D. This article reports the valuation of the CQ-11D in China using online DCE_TTO_ among a representative sample of the Chinese general population.

## Materials and methods

### CQ-11D instrument

Holism based on TCM theories was used to guide the development of the CQ-11D. The methods for formulating the instrument included searching the literature, interviewing patients, consulting experts, and using a questionnaire survey. The original instrument consisted of two parts: a self-rated health status questionnaire and a visual analog instrument score. The self-assessed health status questionnaire had 11 questions (Table 1) and was divided into two sections: ^①^Xing Shen Tong Ju-Xing (physical functioning) Dimension, which centered around physical functioning, contains 8 questions and ^②^Xing Shen Tong Ju-Shen (psychological well-being) Dimension, which centered around psychological wellbeing, contained three questions. According to a preliminary survey of developing CQ-11D, its feasibility evaluation results showed a good acceptance rate. The total Cronbach's α of the scale is 0.820, and the Cronbach's α of each dimension is greater than 0.6, indicating that the instrument had a good internal consistency. Using the exploratory factor analysis method, the KMO value of the scale is 0.791, Bartlett's sphericity test χ2 = 318.414, *P* < 0.05, which is suitable for factor analysis. The factor analysis results showed that the cumulative contribution rate of variance of the three common factors is 58.603%, and the items in the three common factors have the inherent logical relationship of the scale, indicating the instrument had structural validity. The CQ-11D and the EQ-5D-3L as standard benchmark instruments correlated with 0.651, indicating a good standard validity [17].

**Table 1:** Indicators of CQ-11D

Indicator	Index content	Level	Abbreviations
1	Active status and ability to self-care	1	HD1
		2	HD2
		3	HD3
		4	HD4
2	Appetite	1	SY1
		2	SY2
		3	SY3
		4	SY4
3	Stool status	1	DB1
		2	DB2
		3	DB3
		4	DB4
4	Sleep quality	1	SM1
		2	SM2
		3	SM3
		4	SM4
5	Vigor (with vitality, energy, concentration)	1	JS1
		2	JS2
		3	JS3
		4	JS4
6	Dizziness (consciously dizzy, with milder cases closing their eyes and more serious cases unable to stand)	1	TY1
		2	TY2
		3	TY3
		4	TY4
7	Palpitation (conscious heart beating restlessly)	1	XH1
		2	XH2
		3	XH3
		4	XH4
8	Pain	1	TT1
		2	TT2
		3	TT3
		4	TT4
9	Fatigue	1	PL1
		2	PL2
		3	PL3
		4	PL4
10	Irritability	1	FZ1
		2	FZ2
		3	FZ3
		4	FZ4
11	Anxiety (worried, anxious, nervous, worried, uneasy, etc.) or frustrated (disappointed, lack of interest in doing things, no fun, lack of energy, etc.)	1	JL1
		2	JL2
		3	JL3
		4	JL4

### Investigation method and content

The DCE_TTO_ questionnaire was developed by the Lighthouse Studio 9.9.2 software. The accompanying survival time dimensions were set to 4 levels, namely 1 year, 4 years, 7 years, and 10 years. A total of 700 pairs of health conditions were selected and distributed to 70 sets of DCE_TTO_ tasks were generated using the balanced overlap method [27–29]. Each set (i.e., ten DCE_TTO_ tasks) was randomly selected during the survey for the respondent to answer; the task order and the left–right position of health states within each task were all randomized [29]. Mock tests were performed on the generated discrete-choice questionnaires to evaluate the equilibrium of health status extraction. A simulated sample size of 2,400 cases were set in Lighthouse Studio software to test the quality of the discrete choice experimental design. The interaction between each item and the dimension of survival time was checked. The test results showed that 12,015 (50.06%) of the 24,000 choices with a simulated sample size of 2,400 chose option 1, and 11,985 (49.94%) chose option 2. As a general guideline, the standard error should be 0.05 or less for main-effects procedures and 0.10 or less for interaction-effects procedures. The test results show that the standard errors of the main effects are all less than 0.05 (Table 2), the standard errors of the interaction effects are all less than 0.10, and the level of each item is well balanced. Other parts of the questionnaire included CQ-11D, basic information questionnaire, six-dimensional health survey summary form SF-6D, EQ-5D-3L, etc.

**Table 2:** Experimental extraction of equilibrium main effect simulation test results

Indicator	Levels	Decimation frequency	Standard error
1	1	350	0.015
	2	350	0.014
	3	350	0.015
	4	350	0.015
2	1	350	0.015
	2	350	0.016
	3	350	0.015
	4	350	0.015
3	1	350	0.015
	2	351	0.015
	3	349	0.015
	4	350	0.015
4	1	350	0.015
	2	350	0.015
	3	350	0.015
	4	350	0.015
5	1	350	0.015
	2	350	0.015
	3	350	0.015
	4	350	0.015
6	1	350	0.015
	2	350	0.015
	3	350	0.015
	4	350	0.015
7	1	350	0.015
	2	350	0.015
	3	350	0.015
	4	350	0.015
8	1	351	0.015
	2	350	0.015
	3	349	0.015
	4	350	0.015
9	1	350	0.015
	2	350	0.015
	3	350	0.015
	4	350	0.015
10	1	350	0.015
	2	350	0.015
	3	350	0.015
	4	350	0.015
11	1	350	0.015
	2	350	0.015
	3	350	0.015
	4	350	0.015
12	1	350	0.014
	2	350	0.014
	3	350	0.014
	4	350	0.015

items 1–11 correspond to 11 items of the traditional Chinese Medicine quality of Life Assessment scale (CQ-11D), and item 12 represents the dimension of survival time

### Respondent and interviewer

For discrete choice experiments, an average of more than 20 respondents should answer each set of questionnaires in order to estimate a reliable model [30]. The DCE_TTO_ design of this study generated 70 sets of questionnaires, so the effective sample size of this study was planned to be 2,400 respondents. Numerous provinces and cities in mainland China were selected for the investigation. The surveyed provinces and cities spread in North China, Northeast China, East China, Central China, South China, Northwest China, and Southwest China with a total of 28 provinces and municipalities, including 118 prefecture-level cities, to cover sufficient geographical distribution and diversified levels of economic development in China. A stratified sampling method was applied, in which two quotas were set for age and sex, to ensure these distributions of the sample resembled those of the general Chinese population (Table 3) [31]. Recruit participants by posting recruitment advertisements in a way that is convenient for the interviewer. Recruitment was conducted in publicly accessible places (Parks, shops, streets, and university campuses) and private areas (participants’ residences). Respondents are required to meet the following inclusion criteria: ^①^Age ≥ 18 years old; ^②^Chinese citizens with Chinese nationality; ^③^Have been living in Mainland China for the past five years; ^④^Agree to participate in this research. Respondents are also required not to meet the exclusion criteria: ^①^Have listening, speaking, reading, and writing difficulties or are unable to understand the interview content; ^②^Abnormal mental condition. The main steps of the investigation were as follows: ^①^The respondents were screened into the research and informed consent; ^②^The interviewer guided the respondent to complete the CQ-11D questionnaire; ^③^The interviewer guided the respondent in completing the DCE_TTO_ tasks. In addition, after completing the DCE_TTO_ tasks, respondents were asked to self-assess the difficulty of understanding and answering these tasks according to a 5-point Likert scale ranging from very easy to very difficult; ^④^The interviewer guided the respondent to complete the background information questionnaire and the EQ-5D-3L and the SF-6D; ^⑥^Recorded the time for the respondent to complete the survey; ^⑦^Checked whether the questionnaire was clear and complete.

**Table 3:** Sample quota design

Demographic characteristics	Percentage of China's adult population*	Total quota sample size
	**(%)**	***N* = 2400**
***Gender***		
Male	51.13%	1227
Female	48.87%	1173
***Age***		
18-29^a^	20.11%	483
30–39	19.10%	458
40–49	20.28%	487
50–59	18.18%	436
≥ 60	22.32%	536

^*^The proportion of China's adult population was calculated using the data from China Statistical Yearbook (2019);

^a^18–19-year-old population data in the "China Statistical Yearbook (2019)" was included in the 15–19-year-old population, so it was obtained by calculating the average population of each age in the 15–19 years old

### Quality control

A total of 125 interviewers divided into six teams were involved with one quality control leader and one project supervisor in each group. The following quality control methods were carried out.

**Fig. 2:**
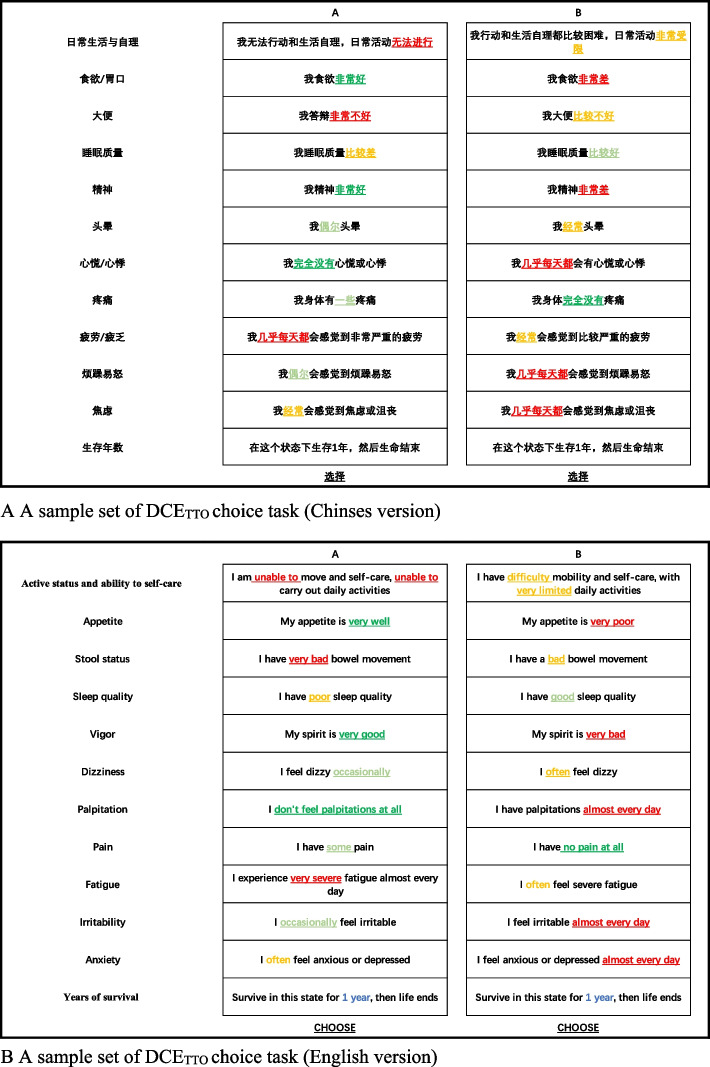
A Sample set of DCETTO choice task (A: Chinses version; B: English version). Note: The corresponding degree words are in the order of best, relatively good, relatively poor, and the worst corresponds to the four colors of dark green, light green, light red, and dark red respectively

1. *Interviewer training.* All interviewers received a full-day training, including DCE operational processes, questionnaire examples, and quality control requirements to ensure equivalent task understanding, standard procedures, and good respondent interactions.
2. *Team management.* All interviewers were divided into six teams. Each team was designated a team leader who was responsible for the management and guidance of interviewers and collecting survey recordings for quality control; there was also a supervisor interviewer who was mainly responsible for the supervision of the process, follow-up visits for respondents, and review of quality control materials (interview sound recordings, informed consents, and other materials) to ensure the data quality.
3. *Questionnaire invalidation criteria*. 1)The respondent had difficulty understanding the task, was impatient, did not cooperate with the interviewer, or did not respond according to relevant requirements and instructions; 2) The interviewer failed to operate in accordance with the research specifications or the interviewer's manual; 3) The respondent failed to complete the entire questionnaire; 4) The time of completing the questionnaire was too short (less than 5 min), which affected the quality of the interview.
4. *The unique design of DCE*_*TTO*_* task choice: *Each item of the DCE_TTO_ task includes four levels, and the corresponding degree words are in the order of best, relatively good, relatively poor, the worst corresponds to the four colors of dark green, light green, light red, and dark red, respectively, in order to facilitate the respondents to understand and remember the degree of the health state (Fig. 2).
5. *Data entry:* Two research team members daily entered and checked the data to ensure accuracy.
6. *Identification of potentially problematic data:* Identified the data who always select the same options, such as “AAAAAAAAAA”; or select “ABABABABAB” in the DCE_TTO_ [16, 32, 33].

### Statistical and analysis methods

The DCE_TTO_ data were analyzed under the random utility framework using a conditional logit model, which assumes a homogenous preference from the respondents, following the model specification proposed by Bansback et al. [16, 19]:

1 $${U}_{i}=\alpha +\beta {t}_{dl}+\sum_{d}\sum_{l}{\lambda }_{dl}{x}_{dl}{t}_{dl}+{\varepsilon }_{i},$$

Among them, U_i_ represented potential utility, t_dl_ represents survival time, x_dl_t_dl_ represented the interaction between item dimension level and survival time, t represented the main effect of survival time, and it was taken as a linear continuous variable [28]. The DCE_TTO_ value for each health state can be anchored on the QALY scale as follows:

2 $${V}_{i}=1+\frac{\lambda }{\beta }{x}_{dl},$$

The variable definitions in the model construction of this study are shown in S1. The dependent variable y is the choice of each respondent, and it is a binary variable with a value of 0 or 1. Independent variables include survival duration, which is considered to be a linear continuous variable. In addition, there are 11 items of the CQ-11D, including “activity” (HD: hd2y, hd3y, hd4y), “appetite” (SY: sy2y, sy3y, sy4y), “Stool status”(DB: db2y, db3y, db4y), “Sleep quality” (SM: sm2y, sm3y, sm4y), “Vigor” (JS: js2y, js3y, js4y), “Dizziness” (TY: ty2y, ty3y, ty4y), “Palpitation” (XH: xh2y, xh3y, xh4y), “Pain” (TT: tt2y, tt3y, tt4y), “Fatigue” (PL: pl2y, pl3y, pl4y), “Irritability” (FZ: fz2y, fz3y, fz4y) and “Frustrated” (JL: jl2y, jl3y, jl4y).

Excel 2016 was used for saving, merging, screening, and basic data conversion. Descriptive statistics were applied by SPSS (Version 20) to summarize the detailed number and proportion of respondents of the specific level of demographic variables. STATA 15.0 was used to construct conditional logit models. We conducted the t-test for continuous variables and the χ2 or Fisher’s exact test for categorical variables. Differences in the distribution of characteristics and model coefficients were considered statistically significant if *p* < 0.05. A correlation coefficient and difference test were used to determine if respondents' responses were consistent and whether health evaluation results differed across instruments. Because of the large sample size in this study, Spearman correlation coefficients and Pearson correlation coefficients are calculated simultaneously in the correlation analysis if the variable does not conform to the normal distribution. For the EQ-5D-3L, the utility value was calculated using the Chinese value set conducted in 2014 [34], and for the SF-6D, the utility value was calculated using the Chinese Hong value set [35, 36].

This study protocol was approved by the ethics committee of the Beijing University of Chinese Medicine (Approval number: 2021BZYLL03012). Informed consent was obtained from all respondents included in the study.

## Results

### Characteristics of the sample

**Fig. 3:**
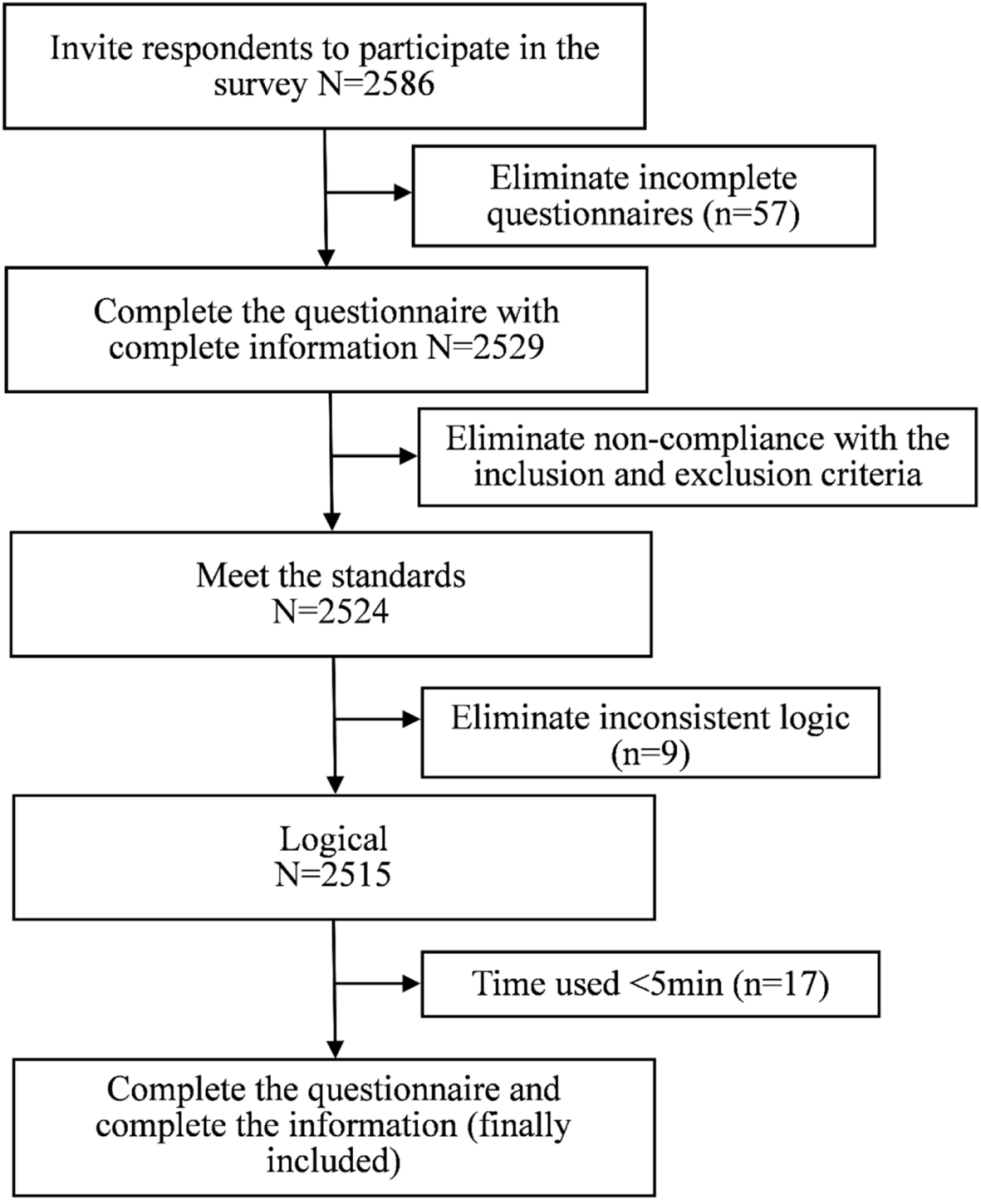
Flow chart of sample inclusion

A total of 2,586 respondents were involved, of which 88 interviews were excluded because the respondents did not complete the whole interview (*N* = 57), or the interviews did not meet the inclusion criteria (*N* = 5), or answered with logical inconsistencies (*N* = 9), or the interview took less than 5 min (*N* = 17). Finally, a total of 2498 respondents were included (Fig. 3). As illustrated in Table 4, 46.08% were males, 42.91% were agricultural accounts, and each geographic distribution ranged from 8.85% to 17.53%. The characteristics of respondents were close to those of the general Chinese population.

**Table 4:** Basic information of respondents

Basic Information	Number	Proportion (%)
**Gender**		
Male	1151	46.08
Female	1347	53.92
**Age**		
18–29	761	30.46
30–39	419	16.77
40–49	548	21.94
50–59	367	14.69
≥ 60	403	16.13
**Nationality**		
Han	2299	92.03
Minority	199	7.97
**Family and marital**		
Unmarried	758	30.34
Married and living together	1603	64.17
Married but separated	22	0.88
Divorced	50	2.00
Widowed	61	2.44
Other	4	0.16
**Education**		
Below elementary school	86	3.44
Primary school	212	8.49
Junior high school	469	18.78
High school or technical secondary	398	15.93
Technical school	345	13.81
Undergraduate	901	36.07
Master and above	87	3.48
**Account type**		
Non-agricultural household	1426	57.09
Agricultural account	1072	42.91
Employment		
Full-time employees	759	30.38
Temporary worker	146	5.84
Hourly worker	4	0.16
Individuals and freelancers	350	14.01
Retired	289	11.57
Student	578	23.14
Farming	211	8.45
Unemployed	149	5.96
Other	12	0.48
**Geographical division**		
North	438	17.53
North-east	349	13.97
East	387	15.49
Central	324	12.97
South	221	8.85
South-west	385	15.41
North-west	394	15.77
**Smoking**		
Never	1831	73.30
Occasionally	231	9.25
Often	360	14.41
Quit	76	3.04
**Alcohol drinking**		
Never	1184	47.40
Occasionally	1104	44.20
Often	172	6.89
Quit	38	1.52
**Physical exercise or fitness activity**		
Often	673	26.94
Sometimes	1472	58.93
Never	353	14.13
**Overall health in the past month**		
Very good	567	22.70
Good	967	38.71
Average	877	35.11
Bad	74	2.96
Very bad	13	0.52
**Changes in health compared to the past year**		
No change	1213	48.56
Got better	586	23.46
Go bad	455	18.21
Not easy to say	244	9.77
**Suffer from chronic diseases**		
Yes	692	27.70
No	1806	72.30
**Average monthly income**		
¥ 0–1300	732	29.30
¥ 1300–3300	653	26.14
¥ 3300–6300	702	28.10
¥ 6300–13000	292	11.69
¥ 13,000–21000	62	2.48
¥ 21,000–42000	37	1.48
¥ 42,000 above	20	0.80
**Have experienced serious health problems**		
Yes	362	14.49
No	2136	85.51

The mean ± SD time of the interviews was 14.5 ± 5.9 min, the minimum was 5.0 min, and the maximum was 52.0 min. 68.29% of the respondents thought that the health status displayed by the DCE_TTO_ tasks was very easy or easy to understand, and 7.65% of the respondents thought it was difficult or very difficult to understand; in terms of tasks choice, 50.56% of the respondents thought it was very easy or easy, and 18.33% of the respondents thought it was difficult or very difficult. Overall, the DCE_TTO_ tasks were relatively easy to complete by the general Chinese population. Nevertheless, potentially problematic answer patterns were observed in respondents who always selected the same options (e.g., 25 respondents responded ‘AAAAAAAAAA’, 5 respondents responded ‘BBBBBBBBBB’ and 6 respondents responded ‘ABABABABAB’) in the DCE_TTO_. These very small proportion of respondents (i.e., 1.40% of total respondents) were not observed noticeable differences in demographic characteristics, and some answers may be due to random errors. Therefore, these respondents were not excluded from this study [28].

### Construction of health utility value set

The results of the conditional logit model estimations are shown in Table 5. There are five non-monotonic coefficients in the conditional logit model, namely SY, SM, TY, PL, and JL. We therefore modified the conditional logit model by combing level 2 with level 1 for the "Appetite " dimension, level 2 with level 1 for the "Sleep Quality" dimension, level 2 with level 1 for the "Dizziness" dimension, level 2 with level 1 for the "Fatigue " dimension, and level 2 and level 1 for the "Anxiety or depression" dimension. The latent utility values generated was then anchored by using the coefficient of the survival time dimension to obtain the anchored coefficients (Table 5).

**Table 5:** Conditional logit model and logit model calculation results

Item level	Conditional logit			Conditional logit (Modified)	
	**coefficient**	**SEM**	***P***	**coefficient**	**hidden utility coefficient**
year	0.270	0.009	< 0.001*	0.287	——
hd2y	-0.023	0.004	< 0.001*	-0.024	-0.083
hd3y	-0.102	0.004	< 0.001*	-0.102	-0.355
hd4y	-0.143	0.004	< 0.001*	-0.144	-0.500
sy2y	**0.011**	0.004	0.005*	0.000	0.000
sy3y	-0.024	0.004	< 0.001*	-0.029	-0.102
sy4y	-0.037	0.004	< 0.001*	-0.043	-0.149
db2y	-0.003	0.004	0.523	-0.003	-0.011
db3y	-0.016	0.004	< 0.001*	-0.017	-0.060
db4y	-0.028	0.004	< 0.001*	-0.028	-0.099
sm2y	**0.009**	0.004	0.032*	0.000	0.000
sm3y	-0.011	0.004	0.005*	-0.015	-0.051
sm4y	-0.030	0.004	< 0.001*	-0.034	-0.118
js2y	-0.005	0.004	0.194	-0.006	-0.022
js3y	-0.022	0.004	< 0.001*	-0.023	-0.079
js4y	-0.041	0.004	< 0.001*	-0.041	-0.143
ty2y	**0.005**	0.004	0.253	0.000	0.000
ty3y	-0.017	0.004	< 0.001*	-0.020	-0.068
ty4y	-0.037	0.004	< 0.001*	-0.039	-0.135
xh2y	-0.002	0.004	0.539	-0.002	-0.007
xh3y	-0.013	0.004	0.002*	-0.013	-0.045
xh4y	-0.038	0.004	< 0.001*	-0.038	-0.131
tt2y	-0.011	0.004	0.006*	-0.010	-0.036
tt3y	-0.033	0.004	< 0.001*	-0.032	-0.112
tt4y	-0.061	0.004	< 0.001*	-0.060	-0.211
pl2y	**0.006**	0.004	0.130	0.000	0.000
pl3y	-0.015	0.004	< 0.001*	-0.017	-0.060
pl4y	-0.030	0.004	< 0.001*	-0.033	-0.114
fz2y	-0.001	0.004	0.738	-0.002	-0.006
fz3y	-0.012	0.004	0.003*	-0.011	-0.040
fz4y	-0.032	0.004	< 0.001*	-0.031	-0.109
jl2y	**0.005**	0.004	0.258	0.000	0.000
jl3y	-0.013	0.004	0.001*	-0.015	-0.052
jl4y	-0.043	0.004	< 0.001*	-0.046	-0.159
Log likelihood	-15,411.18				
AIC	30,890.36				
BIC	31,190.21				

^**^means significant at the level of α = 0.01

*means significant at the level of α = 0.05; AIC represents the Akaike information criterion; BIC represents the Bayesian information criterion; the bolding coefficient is not monotonic

### CQ-11D value set

According to the anchored results of the conditional logit model, it can be determined that the utility value set of CQ-11D based on the health preference of the general population in China was presented in Table 6. The formula for calculating the health utility value of CQ-11D based on the health preference of the Chinese population is as follows:

**Table 6:** CQ-11D utility value set

Item level	Description	Coefficient
Action and life self-care(HD)		
1	I don’t have any difficulty in taking care of myself in my actions and life, and there is no problem in my daily activities	0
2	I have a little difficulty moving, but I can take care of myself, and my daily activities are a little restricted	-0.083
3	I have difficulty taking care of myself in both mobility and life, and my daily activities are very restricted	-0.355
4	I can’t move and take care of myself, and I can’t carry out daily activities	-0.500
Appetite(SY)		
1	My appetite is very good	0
2	My appetite is good	0
3	My appetite is poor	-0.102
4	My appetite is very bad	-0.149
Stool(DB)		
1	My stool movements are very good	0
2	My stool movements are good	-0.011
3	My stool movements are poor	-0.060
4	My stool movements are very bad	-0.099
Sleep quality(SM)		
1	My sleep quality is very good	0
2	My sleep quality is good	0
3	My sleep quality is poor	-0.051
4	My sleep quality is very poor	-0.118
Vigour(JS)		
1	My vigour is very good	0
2	My vigour is good	-0.022
3	My vigour is bad	-0.079
4	My vigour is very bad	-0.143
Dizziness(TY)		
1	I am not dizzy at all	0
2	I occasionally feel dizzy	0
3	I often feel dizzy	-0.068
4	I feel dizzy almost every day	-0.135
Palpitation(XH)		
1	I didn't feel palpitations at all	0
2	I occasionally feel palpitations	-0.007
3	I often feel palpitations	-0.045
4	I feel palpitations almost every day	-0.131
Pain(TT)		
1	I have no pain at all	0
2	I have some pain	-0.036
3	I have severe pain	-0.112
4	I have very severe pain	-0.211
Fatigue(PL)		
1	I don't feel tired at all	0
2	I occasionally feel a little fatigue	0
3	I often feel severe fatigue	-0.060
4	I feel very tired almost every day	-0.114
Irritability(FZ)		
1	I don't feel irritable at all	0
2	I occasionally feel irritable	-0.006
3	I often feel irritable	-0.040
4	I feel irritable almost every day	-0.109
Anxiety or depression(JL)		
1	I don't feel anxious or depressed at all	0
2	I occasionally feel anxious or depressed	0
3	I often feel anxious or depressed	-0.052
4	I feel anxious or depressed almost every day	-0.159

$$\mathrm{U }= 1 - 0.083 \times \mathrm{ HD}2 - 0.355 \times \mathrm{ HD}3 - 0.500 \times \mathrm{ HD}4 - 0 \times \mathrm{ SY}2 - 0.102 \times \mathrm{ SY}3 - 0.149 \times \mathrm{ SY}4 - 0.011 \times \mathrm{ DB}2 - 0.060 \times \mathrm{ DB}3 - 0.099 \times \mathrm{ DB}4 - 0 \times \mathrm{ SM}2 - 0.051 \times \mathrm{ SM}3 - 0.118 \times \mathrm{ SM}4 - 0.022 \times \mathrm{ JS}2 - 0.079 \times \mathrm{ JS}3 - 0.143 \times \mathrm{ JS}4 - 0 \times \mathrm{ TY}2 - 0.068 \times \mathrm{ TY}3 - 0.135 \times \mathrm{ TY}4 - 0.007 \times \mathrm{ XH}2 - 0.045 \times \mathrm{ XH}3 - 0.131 \times \mathrm{ XH}4 - 0.036 \times \mathrm{ TT}2 - 0.112 \times \mathrm{ TT}3 - 0.211 \times \mathrm{ TT}4 - 0 \times \mathrm{ PL}2 - 0.060 \times \mathrm{ PL}3 - 0.114 \times \mathrm{ PL}4 - 0.006 \times \mathrm{ FZ}2 - 0.040 \times \mathrm{ FZ}3 - 0.109 \times \mathrm{ FZ}4 - 0 \times \mathrm{ JL}2 - 0.052 \times \mathrm{ JL}3 - 0.159 \times \mathrm{ JL}4.$$

All the health states described by the CQ-11D can be calculated using this function. For example, if the health status is 11,111,111,111 for all the dimensions, the health utility value is 1. The utility value of the health state "13,112,121,223" can be calculated as U_(13112121223)_ = 1–0-0.102–0-0–0.022–0-0.007–0-0 -0.006–0.052 = 0.811. The worst health state "44,444,444,444" utility value can be calculated as U_(44444444444__)_ = 1–0.500–0.149–0.099–0.118–0.143–0.135–0.131–0.211–0.114–0.109–0.159 = -0.868. The range of measurable utility values of the CQ-11D is -0.868 ~ 1.

### Comparative study on the utility value of CQ-11D, SF-6D, EQ-5D-3L

The correlation analysis results are shown in S2. The utility value measured by the CQ-11D was significantly correlated with the utility value measured by the SF-6D and EQ-5D-3L. As shown in Table 7, the utility value difference between the CQ-11D, SF-6D, and EQ-5D-3L was statistically significant (*P* < 0.01) (S3), and the mean and median utility values of the EQ-5D-3L instrument were the largest, the mean and median utility values of the SF-6D instrument were the smallest, while the mean and median utility values of the CQ-11D were in the middle. Correlation analysis results are shown in S2; the utility value measured by the CQ-11D is significantly correlated with that measured by the SF-6D and EQ-5D-3L. A statistically significant difference (*p* < 0.01) was observed between the CQ-11D, SF-6D, and EQ-5D-3L utility values (S3). Aside from that, EQ-5D-3L had the highest mean and median utility values, the SF-6D had the lowest, and the CQ-11D had a mean and median utility value that was midway between the two.

**Table 7:** Descriptive statistics of health utility values measured by CQ-11D, SF-6D, EQ-5D-3L

Measuring tools	N	Normality test *P*	Mean	SD	Min	Max	Percentile		
							**25**	**50(Median)**	**75**
CQ-11D	2498	< 0.01	0.906	0.118	-0.147	1.000	0.878	0.938	0.976
SF-6D	2498	< 0.01	0.840	0.120	0.346	1.000	0.762	0.873	0.923
EQ-5D-3L	2498	< 0.01	0.930	0.106	0.336	1.000	0.869	1.000	1.000

**Fig. 4:**
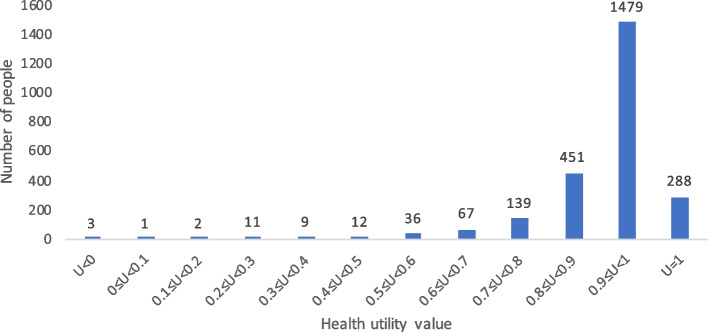
Histogram of the frequency distribution of the health utility value of the general population in China based on CQ-11D

**Fig. 5:**
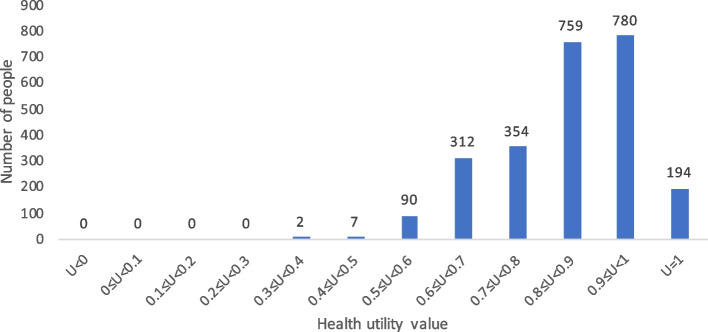
Histogram of the frequency distribution of the health utility value of the general population in China based on SF-6D

**Fig. 6:**
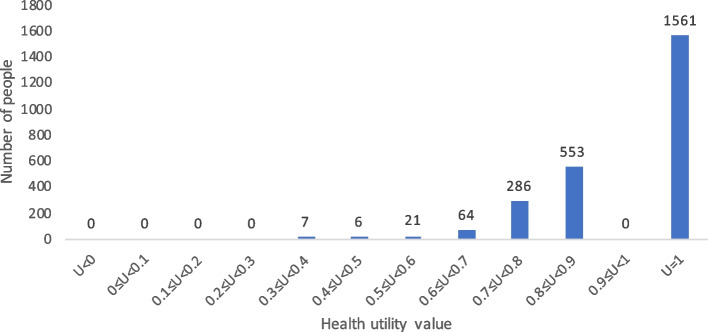
Histogram of the frequency distribution of the health utility value of the general population in China based on EQ-5D-3L measure

When comparing health utility values measured by the CQ-11D, SF-6D, and EQ-5D-3L, the proportions with a health utility value of 1 were 11.53%, 7.77%, and 62.49%, respectively. The specific results are shown in Figs. 4, 5 and 6.

## Discussion

This study reports a Chinese-specific value set for the CQ-11D that can be used for economic evaluations. The measurement range of the value set is -0.868 ~ 1. This study has two substantial advantages. First, the utility of undergoing TCM treatment can be directly quantified for economic evaluation. The CQ-11D can be used to solve the problems of insufficient presentation of PROs and difficult access to health utility values for TCM interventions. It also provides an effective measurement tool for clinical and economic evaluations of TCM. Secondly, it captures aspects of Chinese culture and TCM theory that are not included in other GPBMs (such as appetite, stool, and dizziness). The largest utility decrements in Action and life self-care (HD), Pain (TT), Anxiety or depression (JL), and Appetite (SY) dimensions had more impact on utility values but were not fully reflected in generic instruments.

Even though the utility decrements for the TCM theories-sensitive dimensions were smaller than those for the more generic dimensions, their inclusion provides a more relevant measure of utility for TCM treatment under the Chinese culture. The pits state in this study is -0.868, which is relatively low. Similarly, in the valuation study in Australia for the QLU-C10D, which has a similar number of dimensions to CQ-11D, it was observed that the pits state was -0.96 [37]. Different health state classification systems, valuation methods, and utility functional forms, as well as country-specific cultural disparities in views toward trading between mortality and morbidity, all contribute to variations in the value of health states [38]. One could argue that a lower pits state value indicates a wider range in a utility value set, which might result in more variation between interventions in CUAs [37].

In this study, the DCE_TTO_ questionnaire was developed by the Lighthouse Studio 9.9.2 software. The accompanying survival time dimensions were set to 4 levels, namely 1 year, 4 years, 7 years, and 10 years. A total of 700 pairs of health conditions were selected and distributed to 70 sets of DCE_TTO_ tasks were generated using the balanced overlap method [27–29]. It should be noted that this is not the first time used the lighthouse studio software to develop DCE_TTO_ design. It has been used in previous valuation studies which use DCE or DCETTO [28, 39]. In previous studies, there were some (statistically insignificant) inconsistent coefficients in the DCE_TTO_ model. Following the previous study, the adjacent inconsistent levels were combined when developing value sets, to produce a fully consistent model [28, 32, 40, 41]. Finally, the value set of CQ-11D was generated by the modified conditional logit model of DCE_TTO_ data based on its performance concerning the monotonicity and statistical significance of the coefficients.

The latent utility values generated by the DCE_TTO_ data needs to be rescaled using the coefficient of the additional dimension of survival duration. Compared with traditional approaches such as the standard gamble (SG) and time trade-off (TTO), this approach is easy to understand, operate and manage. The DCE_TTO_ requires respondents to simply point out that option A is better than option B without going through an iterative process to determine at which point the respondents believe that A and B are indistinguishable [19, 42].

Nevertheless, there are certain limitations in that the research process is affected by the understanding and compliance of some populations as a result of the effective sample size included in the age quota. There are more people in the 18–29 age group, and the number of 50–59-year-old participants is relatively small. For a pits state of -0.868, we have some important issues for future research. A related issue is sensitivity to varying degrees of impairment. Assess the sensitivity of CQ-11D to differences in the impact of different degrees (mild or extreme) of QOL and compare them on a generic instrument [37]. There were also some limitations in the experiment design. In this study, the DCE_TTO_ questionnaire was developed by the Lighthouse Studio 9.9.2 software. Sawtooth Software’s procedure does not formally estimate D-efficiency and assumes that designs that are level balanced and near orthogonal will lead to identified preference-model parameters. A disadvantage, however, is that design heterogeneity could be confounded with taste heterogeneity and scale differences [27]. The respondents choose among sets of experimentally controlled sets of profiles and these choices are modeled via multinomial logit as a function of the experimental design variables [29]. However, the multinomial logit model which means it cannot tell the preference heterogeneities of different groups of respondents among the whole sampled population [43]. Thus this study was likely to favor a conditional logit regardless of whether preference heterogeneity was in fact present. However, based on previous studies it was considered necessary to explore the results of mixed logit models [28, 39, 44]. This study explores the results of mixed logit model construction. Since the experiment design bias did not show significant preference heterogeneity, the corresponding results were not presented in the research results section. The relevant results can be found in S4. Another problem is the generate the value set under nonlinear temporal preferences. Jonker et al. find that the best statistical fit was obtained when using a hyperbolic discount function, which resulted in smaller QALY decrements and fewer health states classified as worse than immediate death [45, 46]. It’s unlikely to be able to assess non-linear time preferences in this study given that it was optimized under linear time preferences. In the future, the value set of the CQ-11D can be further improved based on the aforementioned research issues.

## Conclusion

The study provides the first value set for the CQ-11D, which can facilitate cost-utility analyses when applied to data collected with the CQ-11D prospectively and retrospectively. The valuation tool of the CQ-11D was developed for measuring the quality of life and health utility of patients undergoing traditional Chinese medicine interventions. The application of CQ-11D can support TCM resource allocation in China.

### Supplementary Information

**Additional file 1: S1.** Variable settings for model construction. **S2.** CQ-11D utility value and SF-6D, EQ-5D-3L utility value correlation coefficient. **S3.** Tests for differences in utility values of CQ-11D, SF-6D, EQ-5D-3L. **S4.** The results of mixed logit model.

## Data Availability

The datasets used and/or analysed during the current study are available from the corresponding author on reasonable request.
