# Supplementary material for: Valuing Chinese medicine quality of life-11 dimensions (CQ-11D) health states using a discrete choice experiment with survival duration (DCETTO)
Source: Health Qual Life Outcomes. 2023 Aug 23;21:99. doi: 10.1186/s12955-023-02180-4 (PMC10463386; doi:10.1186/s12955-023-02180-4)
Supplement: Supplementary file 1 — Additional file 1: S1. Variable settings for model construction. S2. CQ-11D utility value and SF-6D, EQ-5D-3L utility value correlation coefficient. S3. Tests for differences in utility values of CQ-11D, SF-6D, EQ-5D-3L. S4. The results of mixed logit model. [file 12955_2023_2180_MOESM1_ESM.docx]

**SUPPLYMENTARY MATERIALS**

S1 Variable settings for model construction

| **Variables** | **Meaning** | **Value/Calculation** |
| --- | --- | --- |
| *Dependent variable* |  |  |
| choice | Discrete choice experimental questionnaire selection result, 1 when selected, 0 otherwise | 0、1 |
| *Independent variables* |  |  |
| year | Survival time (considered as a linear continuous variable) | 1、4、7、10 |
| HDk | 1 when the action and life self-care is at k level, 0 otherwise (k=2,3,4) | 0、1 |
| SYk | 1 when the appetite is at k level, 0 otherwise (k=2,3,4) | 0、1 |
| DBk | 1 when the stool is at k level, 0 otherwise (k=2,3,4) | 0、1 |
| SMk | 1 when the sleep quality is at the k level, 0 otherwise (k=2,3,4) | 0、1 |
| JSk | 1 when the vigour is at k level, 0 otherwise (k=2,3,4) | 0、1 |
| TYk | 1 when the dizziness is at k level, 0 otherwise (k=2,3,4) | 0、1 |
| XHk | 1 when the palpitation is at the k level, it is 1; otherwise, it is 0 (k=2,3,4) | 0、1 |
| TTk | 1 when the pain is at the k level, 0 otherwise (k=2,3,4) | 0、1 |
| PLk | 1 when the fatigue is at the k level, 0 otherwise (k=2,3,4) | 0、1 |
| FZk | 1 when the irritable is at the k level, 0 otherwise (k=2,3,4) | 0、1 |
| JLk | 1 when the anxiety is at the k level, 0 otherwise (k=2,3,4) | 0、1 |
| hdky | The interaction between action and life self-care k level and survival time (k=2,3,4) | HDk * year |
| syky | The interaction between appetite k level and survival time (k=2,3,4) | SYk * year |
| dbky | The interaction between stool k level and survival time (k=2,3,4) | DBk * year |
| smky | The interaction between sleep quality k level and survival time (k=2,3,4) | SMk * year |
| jsky | The interaction between vigour k level and survival time (k=2,3,4) | JSk * year |
| tyky | The interaction between dizziness k level and survival time (k=2,3,4) | TYk * year |
| xhky | The interaction between palpitation k level and survival time (k=2,3,4) | XHk * year |
| ttky | The interaction between pain k level and survival time (k=2,3,4) | TTk * year |
| plky | The interaction between fatigue k level and survival time (k=2,3,4) | PLk * year |
| fzky | The interaction between irritability k level and survival time (k=2,3,4) | FZk * year |
| jlky | The interaction between anxiety k level and survival time (k=2,3,4) | JLk * year |

S2 CQ-11D utility value and SF-6D, EQ-5D-3L utility value correlation coefficient

| Measuring tools | Spearman | Pearson | N |
| --- | --- | --- | --- |
| SF-6D | 0.535 ** | 0.512 ** | 2498 |
| EQ-5D-3L | 0.497 ** | 0.531 ** | 2498 |

Note: ** means significant correlation at the level of α=0.01

S3 Tests for differences in utility values of CQ-11D, SF-6D, EQ-5D-3L

| Measuring tools | Mann-Whitney U | *P* | t | *P* | N |
| --- | --- | --- | --- | --- | --- |
| CQ-11D *VS* SF-6D | 1889516.000 | < 0.01 | 28.149 | < 0.01 | 2498 |
| CQ-11D *VS* EQ-5D-3L | 2171364.000 | < 0.01 | -11.079 | < 0.01 | 2498 |
| SF-6D *VS* EQ-5D-3L | 1605155.000 | < 0.01 | -41.394 | < 0.01 | 2498 |

S4 The results of mixed logit model

| **Item level** | **coefficient** | **SEM** | ***P*** |
| --- | --- | --- | --- |
| year | 0.353 | 0.013 | <0.001** |
| hd2y | -0.025 | 0.005 | <0.001** |
| hd3y | -0.123 | 0.005 | <0.001** |
| hd4y | -0.187 | 0.006 | <0.001** |
| sy2y | **0.014** | 0.005 | 0.007** |
| sy3y | -0.032 | 0.005 | <0.001** |
| sy4y | -0.048 | 0.005 | <0.001** |
| db2y | -0.006 | 0.005 | 0.230 |
| db3y | -0.023 | 0.005 | <0.001** |
| db4y | -0.040 | 0.005 | <0.001** |
| sm2y | **-0.041** | 0.012 | 0.001** |
| sm3y | -0.024 | 0.022 | 0.265 |
| sm4y | -0.044 | 0.015 | 0.004** |
| js2y | -0.009 | 0.005 | 0.083 |
| js3y | -0.031 | 0.005 | <0.001** |
| js4y | -0.057 | 0.005 | <0.001** |
| ty2y | **0.006** | 0.005 | 0.194 |
| ty3y | -0.019 | 0.005 | <0.001** |
| ty4y | -0.047 | 0.005 | <0.001** |
| xh2y | -0.004 | 0.005 | 0.403 |
| xh3y | -0.015 | 0.005 | 0.003** |
| xh4y | -0.048 | 0.005 | <0.001** |
| tt2y | -0.014 | 0.005 | 0.006** |
| tt3y | -0.043 | 0.005 | <0.001** |
| tt4y | -0.080 | 0.005 | <0.001* |
| pl2y | **0.005** | 0.005 | 0.305 |
| pl3y | -0.023 | 0.005 | <0.001** |
| pl4y | -0.044 | 0.005 | <0.001** |
| fz2y | -0.001 | 0.005 | 0.777 |
| fz3y | -0.017 | 0.005 | 0.001** |
| fz4y | -0.043 | 0.005 | <0.001** |
| jl2y | **0.005** | 0.005 | 0.343 |
| jl3y | -0.016 | 0.005 | 0.001** |
| jl4y | -0.060 | 0.005 | <0.001** |
| Log likelihood | -14829.79 | |  |
| AIC | 29759.58 | |  |
| BIC | 30200.53 | |  |

Note: ** means significant at the level of α=0.01, * means significant at the level of α=0.05; AIC represents the Akaike information criterion; BIC represents the Bayesian information criterion; the bolding coefficient is not monotonic.
